# A Community Mourn The Death Of Dr. Kuan-Teh Jeang

**DOI:** 10.1186/1423-0127-20-7

**Published:** 2013-02-13

**Authors:** Editorial Board of Journal of Biomedical Science

**Affiliations:** 1 Journal of Biomedical Science, c/o BioMed Central, 236 Gray's Inn Road, London WC1X 8HB United Kingdom

## 

Dear colleagues,

Our loyal friend Dr. Kuan-Teh Jeang passed away unexpectedly on the evening of January 27, 2013. The following night and day an avalanche of emails went across the Retrovirus research community, starting in Australia, then Asia, Europe and America. Great shock and sorrow was apparent in the messages by the very many colleagues with whom Teh interacted over the years. Many of us came to know Teh (Additional file 1) as an energetic and gifted scientist for whom we had much respect and affection.

Teh was chief of the Molecular Virology Section in the Laboratory of Molecular Microbiology at the National Institutes of Health in Bethesda, USA. His major research interest was around the human immunodeficiency virus (HIV-1) and human T-cell leukemia virus (HTLV-I), with an abundant production of more than 300 scientific publications on the molecular details of virus replication and the disease-causing mechanisms. He trained over 30 postdoctoral fellows and had been a fantastic mentor of young scientists who have since spread across the globe, from Taipei to Hong Kong, from Montpellier to Amsterdam and from Montreal to Los Angeles.

Teh was a very dynamic and internationally oriented scientist and he always felt a special bond with the scientific community in Taiwan. He was born and spent his early childhood in Taiwan, and later learned to speak Mandarin fluently. He actively participated in the scientific review processes in the National Science Council and the National Health Research Institute and various universities, often travelling long distances to Taiwan to offer comments and suggestions on various aspects of science. He had deep affection towards his home land Taiwan and hence contacted the founding editor Prof. C.C. Chang and volunteered his service for the Journal of Biomedical Science (JBS) of the National Science Council of Taiwan, which was the only international journal sponsored by Taiwan government at that time. He joined the editorial board of JBS in 1994 and had been very influential since then. Teh was a stabilizing force during the formative years of JBS when the biomedical community in Taiwan was somewhat skeptical of an international journal with its Editorial Office based in Taiwan. After JBS was listed in the Journal citation Index in 1999, he was also an avid advocate for ways to improve its impact factor. It is largely due to his suggestions and sometimes unorthodox ideas that JBS jumped in the annual Impact Factor ranking. The creation of Vignettes, which was well-received by our readers, was one of those suggestions.

He left our board in 2004 to free himself for an important new activity: the launch of the Retrovirology journal, where he had been editor-in-chief since then. Again his talent to kick off new initiatives paid off and Retrovirology is currently among the highest cited journals in the field of virology, all achieved in less than 10 years. Since the early years, he had been an advocate of the Open Access publishing format. He was invited for the JBS editorial board meeting last September to present his current views on this topic and gave his most insightful suggestions. Teh always kept a special interest in Taiwan and its research activities, which led to several productive collaborations. He was also a champion for advocacy of the rights of Taiwanese scientists in international scientific organizations. He was elected as Academician of the Academia Sinica in 2008.

Teh was a scientist with a vision and a broad interest in all aspects of the scientific endeavors. He also was a true scientific leader, starting scientific debate, writing editorials, sitting on many committees, orchestrating new book volumes and organizing international meetings on diverse topics. For instance, he was president of the Society of Chinese Bioscientists in America (SCBA) in 2010 and voiced a strong opinion to increase the representation of Asian-American scientists in leadership positions.

Teh's death is a blow to the retrovirus research community and we will sorely miss his scientific leadership. He has been central to so much of what we have done together as well as being a supportive and generous friend to many of us individually. In his spirit, several initiatives have been proposed over the last couple of days by his students and colleagues to honor his legacy. Teh was only 54 years old and the loss is devastating for the Jeang family. Our thoughts and condolences are with his wife Diane and his children David, John and Diana.

Present and past members of the Editorial Board

Journal of Biomedical Science

## Supplementary Material

Additional file 1 Dr. Kuan-Teh Jeang. Click here for file

