# Supplementary figures and images for: A Community Mourn The Death Of Dr. Kuan-Teh Jeang
Source: J Biomed Sci. 2013 Feb 13;20(1):7. doi: 10.1186/1423-0127-20-7 (PMC3599596; doi:10.1186/1423-0127-20-7)

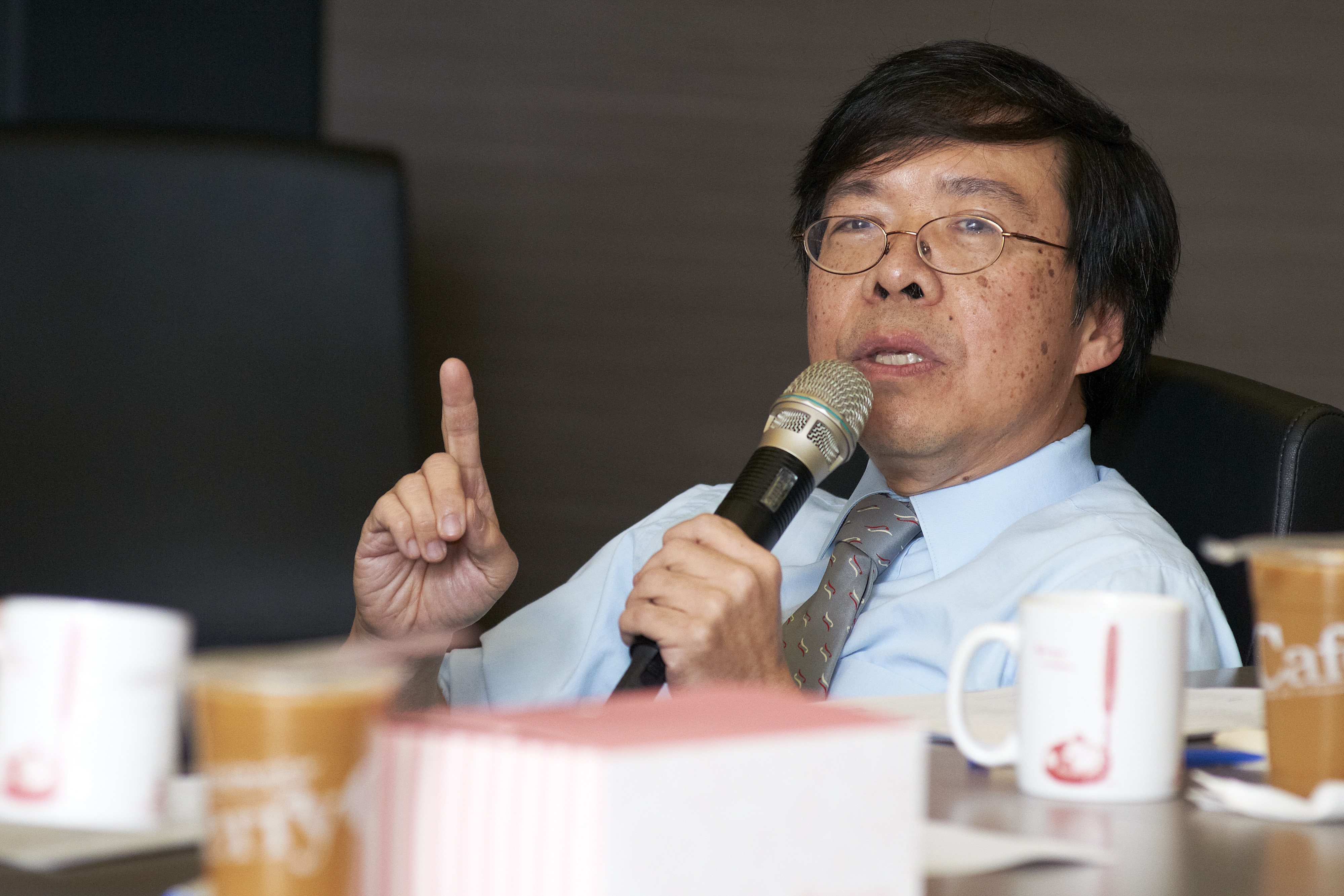

Supplement: Additional file 1 — Dr. Kuan-Teh Jeang. [file 1423-0127-20-7-S1.jpeg]
